# *Cryptoporus volvatus* Extract Inhibits Influenza Virus Replication *In Vitro* and *In Vivo*

**DOI:** 10.1371/journal.pone.0113604

**Published:** 2014-12-01

**Authors:** Li Gao, Yipeng Sun, Jianyong Si, Jinhua Liu, Guibo Sun, Xiaobo Sun, Li Cao

**Affiliations:** 1 Institute of Medicinal Plant Development, Chinese Academy of Medical Sciences and Peking Union Medical College, Beijing, China; 2 Key Laboratory of Animal Epidemiology and Zoonosis, Ministry of Agriculture, College of Veterinary Medicine, China Agricultural University, Beijing, China; University of North Carolina School of Medicine, United States of America

## Abstract

Influenza virus is the cause of significant morbidity and mortality, posing a serious health threat worldwide. Here, we evaluated the antiviral activities of *Cryptoporus volvatus* extract on influenza virus infection. Our results demonstrated that the *Cryptoporus volvatus* extract inhibited different influenza virus strain replication in MDCK cells. Time course analysis indicated that the extract exerted its inhibition at earlier and late stages in the replication cycle of influenza virus. Subsequently, we confirmed that the extract suppressed virus internalization into and released from cells. Moreover, the extract significantly reduced H1N1/09 influenza virus load in lungs and dramatically decreased lung lesions in mice. And most importantly, the extract protected mice from lethal challenge with H1N1/09 influenza virus. Our results suggest that the *Cryptoporus volvatus* extract could be a potential candidate for the development of a new anti-influenza virus therapy.

## Introduction

Influenza is a serious public health problem that causes severe illnesses and deaths for higher risk populations. Influenza A viruses are responsible for seasonal epidemics and have caused three pandemics in the 20^th^ century (1918, 1957, and 1968) as well as the 2009 H1N1 pandemic. Annually, up to 10% of the U.S. population is affected by symptomatic influenza infection. It had been reported that more than 220,000 persons are hospitalized, of which 24,000 die due to influenza-associated illness each year [1]. The highest hospitalization rate occurs in aged population, children and young persons, about one per 1000 or higher in infants, persons at age 65 (approx. 20% of deaths) and older as well as persons with chronic medical conditions [2].

Influenza virus, which is an enveloped, negative-strand RNA virus with a segmented RNA genome, is characterized by frequent mutations - antigenic drifts (minor antigenic change, both A and B) and antigenic shifts (major antigenic change, only A). To combat the virus, a number of treatments are currently available. Vaccines, such as cell-based whole-virion inactivated vaccines and dose-sparing adjuvants [3], [4], [5], can provide prophylactic protection by stimulating the production of antibodies to viral strains. However, these vaccines generally have lower efficacy in the most susceptible populations, such as the elderly over 61 years of age and children less than 11 years of age [6]. Antiviral agents can be used in either a therapeutic or a prophylactic mode. These antiviral agents include M2 ion channel blockers (amantadine and rimantadine) and neuraminidase inhibitors (oseltamivir, zanamivir, and peraivir) [7], [8]. However, the potential usefulness of M2 ion channel blockers is limited due to their lack of activity against influenza B virus, the global distribution of amantadine-resistant influenza A viruses, and the occurrence of neurological side effects [9]. The neuraminidase inhibitor resistance is also mounting with continued use. Oseltamivir-resistant mutants in A/H3N2- and A/H5N1-infected patients receiving this drug have been isolated [10], [11], and oseltamivir-resistant A/H1N1 strains have worldwide spread (during the period 2007 to 2009). Therefore, novel antiviral agents are in urgent need to prepare for future influenza epidemics and pandemics.

Natural products can be candidates to be identified as new generations of antivirals [12]. The medicinal use of mushrooms has a very long tradition in the Asian countries, and their use in the Western hemisphere has been slightly increasing since the last decades [13], [14], [15], [16]. Antiviral effects are described not only for whole extracts of mushrooms [17] but also for their isolated compounds [18], [19]. The antiviral activity could be caused directly by inhibition of viral enzymes, synthesis of viral nucleic acids, or adsorption and uptake of viruses into mammalian cells. These direct antiviral effects are exhibited especially by smaller molecules. Indirect antiviral effects are the result of the immunostimulating activity of polysaccharides or other complex molecules [20]. *Cryptoporus volvatus* belongs to *Eumycota*, *Cryptoporus* [21], and grows in certain areas in China. Its fruiting body has been used for asthma and bronchitis back to the 15^th^ century a.d. when the record of *Cryptoporus volvatus* appeared in “Materia Medica of Yunnan” [22]. Chemical analysis of *Cryptoporus volvatus* reveals that it contains many physiological activators, such as polysaccharose, amino acid, volatile oil, and cryptoporic acids etc. [23]. Aqueous extract from the fruiting body of *Cryptoporus volvatus* has been reported to have anti-tumor, anti-allergy, anti-inflammation, and immunomodulatory activities [24], [25], [26].

We previously showed that the aqueous extract from the fruiting body of *Cryptoporus volvatus* has potential antiviral effects against porcine reproductive and respiratory syndrome virus (PRRSV) infection *in vivo* and *in vitro* [27]. In the present study, we investigated whether aqueous extract from the fruiting body of *Cryptoporus volvatus* has the ability to inhibit influenza virus infection. We first examined its potential to inhibit different influenza virus strain replication *in vitro*, and then determined if the extract could protect mice from lethal challenge with 2009 pandemic H1N1 influenza virus. Our results showed that the extract from *Cryptoporus volvatus* inhibited influenza virus infections *in vitro* through targeting an early stage in the replication cycle, very likely the virus entry into host cells, and the release of the virus from cells. More importantly, *Cryptoporus volvatus* efficiently inhibited 2009 pandemic H1N1 influenza virus *in vivo*. These results implicate that the aqueous extract from the fruiting body of *Cryptoporus volvatus* has the potential to be an antiviral therapeutics against influenza virus infection.

## Materials and Methods

### Ethics statement

All animal research was approved by the Beijing Association for Science and Technology (approval ID SYXK (Beijing) 2007–0023) and complied with the guidelines of Beijing Laboratory Animal Welfare and Ethics of the Beijing Administration Committee of Laboratory Animals.

### Cells and viruses

Madin-Darby Canine Kidney (MDCK) cells were maintained in DMEM supplemented with 10% FBS and penicillin/streptomycin.

The pandemic H1N1/2009 influenza virus, A/Beijing/7/2009(H1N1/09), was isolated from a young patient with an influenza-like illness in December 2009 [28]. Influenza A viruses A/WSN/33(H1N1), A/Jiangxi/262/05(H3N2), and H1N1/09 were grown and titrated on MDCK cells and then stored at −80°C. Briefly, virus was serially diluted 10-fold in DMEM to infect MDCK cells in 96-well plates. Influenza virus infection was determined 36 h post infection using immunofluorescent staining for the virus NP protein. Virus titer was determined using Reed-Muench method, and expressed as tissue culture infective dose 50% (TCID_50_).

### Indirect immunofluorescence assay

Cells were fixed with cold methanol-acetone (1∶1) for 10 min at 4°C, washed with phosphate-buffered saline (PBS), and then blocked with 5% normal goat serum for 30 min at room temperature. After blocking, cells were stained with mouse monoclonal antibody AA5H (Abcam, Hong Kong) against influenza A virus NP. Cells were then washed and incubated with FITC-conjugated goat anti-mouse IgG (H+L) (1∶2000, Jackson ImmunoResearch) for 60 min at 37°C. After three washes in PBS, cells were counter-stained with DAPI and examined by fluorescence microscopy.

### Preparation of the *Cryptoporus volvatus* extract

The *Cryptoporus volvatus* was purchased from Yunnan Province, China. The dry fruiting body of *Cryptoporus volvatus* was crushed by grinder and soaked in distilled water (1 g dry fruiting body in 20 ml H_2_O) overnight at 4°C, and then centrifuged at 8000–10,000 g for 30 min. The supernatant was harvested and freeze-dried, and then stored at −80°C until use. When used, the freeze-dried powder was re-dissolved with normal saline or culture medium and filtered with 0.22 mm filters. The final concentration was determined by the weight of the dry fruiting body and the volume of solvent finally used,

### Cell viability assay

The MTT [3-(4,5-dimethyl-2-thiazolyl)-2,5-diphenyl-2H-tetrazo-lium bromide] assay was used to examine the effect of the *Cryptoporus volvatus* extract on cell viability. MDCK cells in 96-well plates were treated with sequential dilutions of the extract or normal saline in a total of 100 µl growth medium for 48 h. And then, 20 µl of freshly made 5 mg/ml MTT solution was added to each well, and the cells were incubated at 37°C for another 5 h before the medium was replaced with 200 µl DMSO to dissolve the crystals. The plates were further incubated at 37°C for 5 min to dissolve any air bubbles before the MTT signal was measured at an absorbance of 550 nm. The 50% cytotoxic concentrations (CC_50_) were analyzed by GraphPad Prism (GraphPad Software, San Diego, CA).

### Inhibition of virus infection assay

Confluent MDCK cell monolayers in 96-well plate were inoculated with the different influenza virus (multiplicity of infection [MOI]  = 0.1) in the presence of different concentrations of the *Cryptoporus volvatus* extract and 2 mg/ml TPCK-treated trypsin. Twenty-four hours later, the supernatant was collected for virus titration and cells were fixed for indirect immunofluorescence assay. The 50% effective concentration (EC_50_) was determined using a 4 parameter, nonlinear regression of dose response inhibition by plotting log (inhibitor(concentration) vs. viral titer (variable slope) using GraphPad Prism (GraphPad Software, San Diego, CA).

### Time-of-addition experiment

Confluent MDCK cell monolayers in 96-well plate were inoculated with influenza virus A/WSN at an MOI of 1 at 4°C for 2 h and then shifted to 37°C (this time point was set up as 0 h). And the extract was added at −1 h, 0 h, 1 h, 2 h, 4 h, 6 h or 8 h p.i.. At 9 h p.i., the supernatants were collected for virus titration.

### Determination of direct virion inactivation activity of the *Cryptoporus volvatus* extract

Influenza virus A/WSN of 10^5^ TCID_50_ was incubated with different concentrations of the *Cryptoporus volvatus* extract for 1 h or 3 h at 37°C. Following the treatment, viral infectivity was determined on MDCK cells.

### Virus attachment assay

MDCK cells were incubated with different concentrations of the *Cryptoporus volvatus* extract and A/WSN (MOI of 2) at 4°C for 2 h. After cells were washed 6 times with cold PBS, cell lysates were prepared by rapid freeze-thaw. Virus titer was determined as described above.

### Virus entry assay

MDCK cells in 6-well plates were infected with influenza virus at an MOI of 10 at 4°C for 1 h. The inoculum was aspirated, and the cell monolayer was washed three times with cold PBS, replaced with fresh DMEM medium containing different concentration of the extract, and switched to 37°C. At 1 h post switching to 37°C, cells were washed twice with acidic PBS-HCl (pH1.3) to remove any un-internalized viral particles on cell surface, followed by washing twice with PBS. Intracellular virus was analyzed using quantitative RT-PCR. Total cellular RNAs were extracted using Trizol Reagent (Invitrogen) and reverse transcription (RT) was conducted using oligonucleotides specific for vRNA (5′-AGCAAAAGCAGG-3′). A (GAPDH)-specific primer (5′- GAAGATGGTGATGGGATTTC-3′) was also included in the RT reaction mixture. The quantitative real-time PCR was carried out with a 20 µl reaction mixture containing primers specific for influenza virus M1 gene (5′-ACAGATTGCTGACTCCCAGC-3′ and 5′-TCTCATCGCCTGCACCATTT-3′) or for GAPDH RNA (5′-GAAGGTGAAGGTCGGAGTC-3′ and 5′-GAAGATGGTGATGGGATTTC-3′) by using SYBR green DNA dye (TAKARA) following the manufacturer's introductions.

### Virus Release Assay

MDCK cells were infected with A/WSN (MOI = 0.1). At 10 h p.i., cells were washed 3 times with PBS and replaced with fresh medium containing different concentrations of the *Cryptoporus volvatus* extract or BFA (1 mg/ml). At 0.5 h and 1 h following medium replacement, supernatants were collected and cells were lysed by rapid freeze-thaw on dry ice-ethanol and a 37°C water bath. Influenza virus RNA copies in the supernatants (extracellular virus) and cell lysates (intracellular virus) were then quantified using quantitative real-time PCR as described above.

### Treatment studies in mice

Six-week-old female BALB/c mice (Vital River Laboratories [VRL], China) were used in five treatment groups, and mice were anaesthetized with Zoletil (tiletamine-zolazepam; Virbac S.A., Carros, France; 20 µg/g body weight) before treatment. Two groups of mice were intranasally inoculated with 10^3.5^ pfu BJ09 H1N1 virus and then treated with the *Cryptoporus volvatus* extract (16.5 µg extract/g body weight or 50 µg extract/g body weight, respectively). Treatment was given twice daily for 8 days post virus inoculation (dpi) with half of the dosage *via* intragastric administration and the other half *via* thigh muscle injection. One group of mice was administered with the extract (50 µg extract/g body weight) without virus inoculation. One group of mice inoculated with BJ09 H1N1 virus was provided twice daily with normal saline, and one group of mice was just provided with normal saline. At 1, 4, and 7 dpi, three mice in BJ09/normal saline, or BJ09/50 µg/g treatment groups were euthanized and the lungs were collected for virus detection and titration. Also, at 4 dpi, lung sections from three mice in BJ09/normal saline, BJ09/16.5 µg/g, or BJ09/50 µg/g treatment group were collected for immunohistological analysis. Five mice in each group were monitored daily for 14 days for clinical signs post inoculation. Any mouse that lost >25% of its pre-inoculation body weight was euthanized.

### Histopathological analyses

The lung sections were fixed in 10% (wt/vol) buffered formalin. Formalin-fixed tissues were then embedded in paraffin wax, sectioned into 5-mm slices, and mounted on glass slides. Tissues were stained with hematoxylin and eosin (H&E) for light microscopy, or stained for viral NP by immunohistochemistry (IHC) with a mouse monoclonal antibody as previously described [29]. Lung pathology was scored on a scale of 0 to 4 as previously described [29], [30]. Briefly, four easily identifiable pathological processes were chosen to be scored on a scale of 0–4: alveolar and interstitial edema; haemorrhage; margination; and infiltration of inflammatory cells and formation of bronchiolitis. A score of 0 represented normal lungs; a score of 1 represents mild (<25% lung involvement); a score of 2 represents moderate (25–50% lung involvement); a score of 3 represents severe (50–75% lung involvement); and a score of 4 represents the severest (>75% lung involvement). The results of histopathological changes were expressed as mean +SD (five lung sections from each mouse, and 3 mice per group).

### Statistical analysis

Results were analyzed using *One-way ANOVA* except for the animal experiment, which was analyzed using *Student*'*s t test*. Differences were considered to be statistically significant if the *P* value is less than 0.05. *P<0.05; **P<0.01.

## Results

### *Cryptoporus volvatus* extract inhibits influenza virus infection *in vitro*

**Figure 1:**
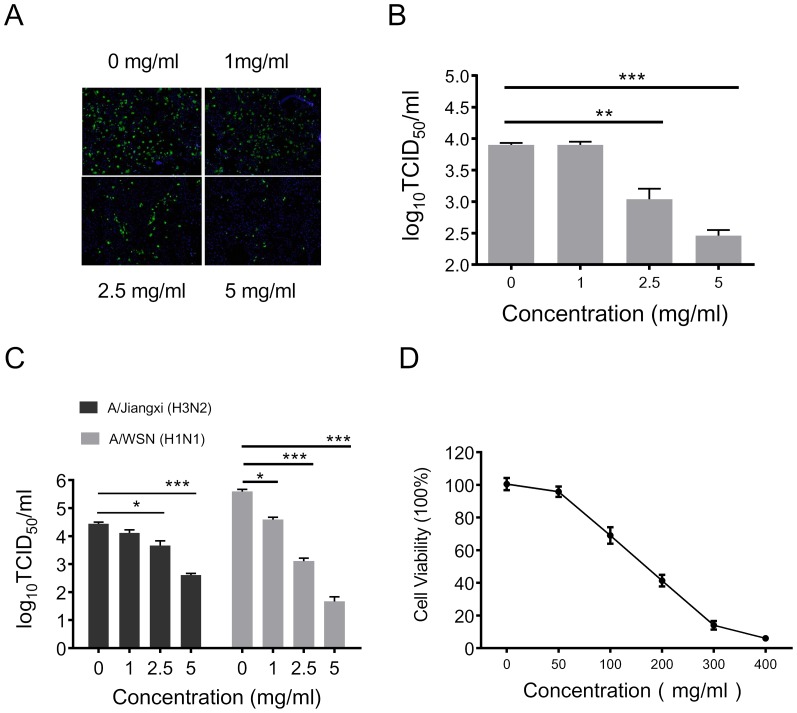
*Cryptoporus volvatus* extract inhibits influenza virus infection *in vitro*. (A and B) The *Cryptoporus volvatus* extract represses H1N1/BJ09 replication in MDCK cells. MDCK cells were infected with H1N1/BJ09 at an MOI of 0.1, and then treated with the *Cryptoporus volvatus* extract at various concentrations or the control normal saline. At 24 h p.i., cells were fixed and analyzed by IFA using antibody against virus NP protein (A), and virus yield in the supernatants was also quantified (B). Cultures treated with normal saline were set up as control (0 mg/ml). (C) *Cryptoporus volvatus* extract potently inhibits both A/Jiangxi (H3N2) and A/WSN (H1N1) replication in MDCK cells. A similar virus inhibition assay was performed with MDCK cells infected with A/Jiangxi (H3N2) or A/WSN (H1N1) at an MOI of 0.1 in the presence of the *Cryptoporus volvatus* extract at various concentrations or the control normal saline. (D) Determination of cytotoxicity of the *Cryptoporus volvatus* extract by MTT assay. MDCK cells were incubated with various concentrations of the *Cryptoporus volvatus* extract or the control normal saline for 48 h prior to the MTT assay. Data are representative of three independent experiments (mean ±SEM). Statistical significance was analyzed by *One-way ANOVA.* *P<0.05; **P<0.01.

To evaluate the therapeutic potential of the *Cryptoporus volvatus* extract, we first investigated whether the extract could inhibit influenza virus replication *in vitro*. Madin-Daby canine kidney (MDCK) cells were inoculated with A/Beijing/7/2009 H1N1 (H1N1/09) influenza virus and then treated with the extract at different concentrations. Twenty-four hours post infection, infected cells were directly observed under microscope following immunofluorescence staining of the virus (Fig. 1A) and the virus quantity in supernatants for each treatment was measured (Fig. 1B). Our results showed that the *Cryptoporus volvatus* extract treatment induced a significant dose-dependent reduction of infected cells and suppressed the propagation of the H1N1/09 virus to a low level (about 7-fold and 30-fold reduction when treated with 2.5 mg/ml, and 5 mg/ml extract, respectively). To further verify its anti-influenza virus activity, we examined whether the *Cryptoporus volvatus* extract could inhibit different influenza virus strain replication in MDCK cells. As illustrated in Figure 1C, the *Cryptoporus volvatus* extract also inhibited seasonal influenza virus A/Jiangxi/262/05(H3N2) and laboratory-adapted A/WSN/33 H1N1 (A/WSN) infections. Virus titer was decreased about 60-fold for H3N2 when the extract was at 5 mg/ml. For A/WSN, the suppression even reached to 10^4^ fold with the extract at the concentration of 5 mg/ml. The extract inhibited influenza virus infection with 50% effective concentration (EC50) values of 0.45 mg/ml for H1N1/09 strain, 1.21 mg/ml for H3N2 strain, and 0.37 mg/ml for A/WSN strain.

To exclude the possibility that nonspecific toxicity induced by the extract could affect influenza virus replication, we evaluated MDCK cell viability under various concentrations of the *Cryptoporus volvatus* extract using the MTT assay (Fig. 1D). Forty-eight hours following treatment, the cells cultured in medium containing 50 mg/ml *Cryptoporus volvatus* retained approximately relative viability of 100% compared with control. And the relative viability of MDCK cells was reduced to less than 10% when the *Cryptoporus volvatus* extract concentration in medium was 400 mg/ml. The 50% cytotoxic concentration (CC50) of the *Cryptoporus volvatus* extract for MDCK cells was 148 mg/ml, which greatly exceeded its EC50. The therapeutic index (CC50/EC50) in MDCK cells was 328 for H1N1/09 virus strain,68 for H3N2 virus strain, and 400 for A/WSN virus strain.

These results suggested that the *Cryptoporus volvatus* extract could inhibit influenza virus infection *in vitro*.

### *Cryptoporus volvatus* extract acts at early and late stages in the replication cycle

**Figure 2:**
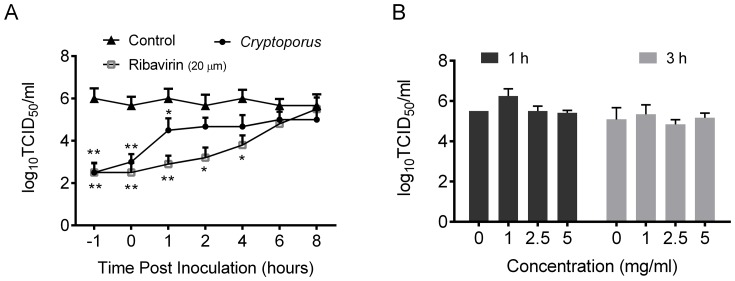
*Cryptoporus volvatus* extract acts at an early and late stages in the replication cycle. (A) Time course analysis of the extract inhibitory effects on influenza A virus replication. MDCK cells were infected with A/WSN (H1N1) at an MOI of 1, and then at different time points, treated with the normal saline control, extract (5 mg/ml), or ribavirin (20 µm). Virus titer at 9 hpi was determined. (B) The *Cryptoporus volvatus* extract did not inactivate influenza virus directly. Incubating the A/WSN (H1N1) virus with different concentrations of the extract at 37°C for 1 h or 3 h, then virus titer was determined in MDCK cells. Data are representative of three independent experiments (mean ±SEM). Statistical significance was analyzed by *One-way ANOVA*. *P<0.05; **P<0.01.

To characterize the specific step(s) of the influenza virus life cycle that is inhibited by the *Cryptoporus volvatus* extract, we examined the time course of their inhibitory effects. MDCK cells were infected with A/WSN virus at an MOI of 1 and, then treated with the extract at various time points post infection from -1 h to 8 h p.i.. We then measured the titer of infectious viral particles released into the supernatant at 9 h p.i. [31]. As shown in Figure 2A, when the *Cryptoporus volvatus* extract was added at −1 h p.i., virus production was strongly blocked by a factor of about 3000 folds, while the extract displayed partial antiviral effect when added at 1 h p.i. or later to 8 h p.i., suggesting that the *Cryptoporus volvatus* extract is able to inhibit earlier and late stages in the virus replication cycle. However, for ribavirin which inhibits virus RNA synthesis, virus production was continuously inhibited between 1 h and 8 h p.i. as a function of compound addition. To verify whether the *Cryptoporus volvatus* extract can directly inactivate the virus infectivity, we incubated A/WSN (H1N1) virus with the *Cryptoporus volvatus* extract at 37°C for 1 h or 3 h. As shown in Fig. 2B, incubation of A/WSN (H1N1) virus with the *Cryptoporus volvatus* extract had no effect on viral infectivity, suggesting that the inhibitory effect of the *Cryptoporus volvatus* extract is not due to its direct inactivation of H1N1 virion particles.

### *Cryptoporus volvatus* extract blocks influenza virus entry into cells

Early events of influenza virus infection cycle include virus attachment and cell entry. Thus, we investigated whether the *Cryptoporus volvatus* extract could block virus attachment or entry into host cells.

**Figure 3:**
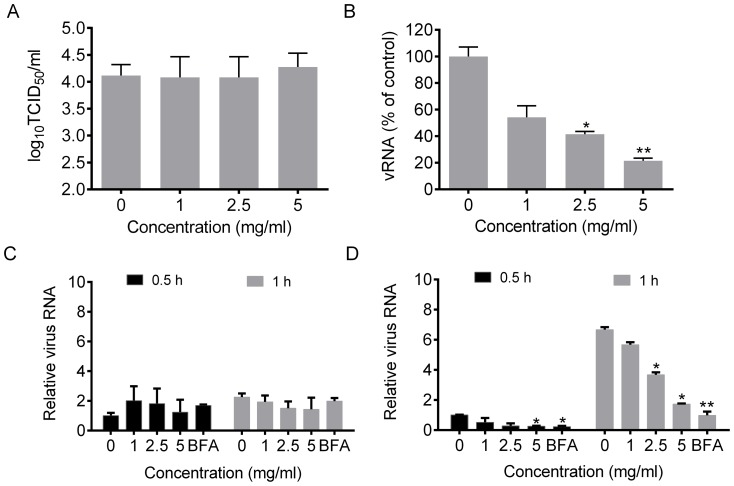
*Cryptoporus volvatus* extract inhibits influenza virus entry into and release from MDCK cells but not attachment. (A) Effects of the *Cryptoporus volvatus* extract on virus attachment. MDCK cells were incubated with various concentrations of the *Cryptoporus volvatus* extract and A/WSN (MOI of 2) at 4°C for 2 h. After cells were washed 6 times with PBS to remove unattached virus, cell lysates were prepared by rapid freeze thaw. Virus titer was determined in MDCK cells. (B) Inhibition of influenza virus entry by the *Cryptoporus volvatus* extract. MDCK cells were incubated with A/WSN (MOI of 10) at 4°C for 2 h. The inoculum was then aspirated, and cell monolayer was washed with cold PBS, replaced with fresh DMEM medium containing various concentrations of the extract or normal saline control, and switched the temperature to 37°C. At 1 h post switching to 37°C, cells were washed twice with acidic PBS-HCl (pH1.3) to remove any un-internalized viral particles on the cell surface, followed by washing twice with PBS. Intracelluar virus was analyzed by quantified M1 gene using Real-Time RT-PCR and the quantity of virus in the control group was set up at 100%. Determination of viral RNA intracellular (C) or released to the supernatants (D). MDCK cells were infected with A/WSN (MOI of 0.1) for 10 h. After washing with PBS, cells were treated with either *Cryptoporus volvatus* extract or BFA (a known inhibitor of protein transport) for 0.5 or 1 h, and then copies of viral RNA in the supernatants and in the cells were determined using quantitative real-time RT-PCR assay. The relative quantities of viral RNA compared to the control at 0.5 h post treatment (set up as 1) were shown. Results shown are the averages from three independent experiments (mean ±SEM). Statistical significance was analyzed by *One-way ANOVA.* *P<0.05; **P<0.01.

We first determined the role of the *Cryptoporus volvatus* extract in virus attachment. As shown in Fig. 3A, the *Cryptoporus volvatus* extract did not affect the quantity of infectious virus particles that can attach to host membranes. To test whether the *Cryptoporus volvatus* extract acts at the internalization stage of infection, we used an assay described before [32]. Influenza viruses were allowed to bind MDCK cells at 4°C for 1 h. Then the inoculum was replaced with fresh DMEM medium containing the *Cryptoporus volvatus* extract, and the cells were transferred to 37°C. Cell-attached viruses started to enter cells through endocytosis when the temperature was shifted from 4°C to 37°C. At 1 h post switching to 37°C, cells were washed with acidic PBS-HCl (pH = 1.3) to remove any un-internalized viral particles on the cell surface, and intracellular virus was analyzed using Real-Time PCR for the M1 gene. As illustrated in Fig. 3B, intracellular uptake of influenza virus was dose-dependently blocked by the *Cryptoporus volvatus* extract, and only about 20% virus entry into cells compared of control was detected when the extract was at 5 mg/ml.

### *Cryptoporus volvatus* extract inhibits the release of influenza virus particles

To investigate whether the *Cryptoporus volvatus* extract inhibits virus release, we used an assay described previously [31] to quantify viruses that are either in cells or released into the supernatants. We first infected MDCK cells with A/WSN (MOI = 0.1), and at 10 hours post infection, cells were then extensively washed with PBS and replaced with fresh medium containing different concentrations of the extract or BFA (1 mg/ml), a known inhibitor of protein transport [33]. Viral RNA copies that were either in cells or released into the supernatants were then quantified at 0.5 h and 1 h following treatments. At each time point, comparable amounts of intracellular viral RNAs were found in either extract or BFA-treated samples (Fig. 3C). In contrast, the relative copies of released viral RNA in supernatants were significantly decreased by 70% when treated with the extract at the concentration of 5 mg/ml compared to the control. And the extract at the concentration of 2.5 mg/ml also potently blocked virus release by about 50% compared to the control at 1 h following treatment (Fig. 3D). There was no significant effects observed when the extract was at 1 mg/ml. It should be noted that we analyzed the released viral RNA, which cannot exactly represent the released virus particles. Nevertheless, our data suggest that the extract block influenza virus particle release.

### *Cryptoporus volvatus* extract inhibits H1N1/09 influenza virus infection *in vivo*

**Figure 4:**
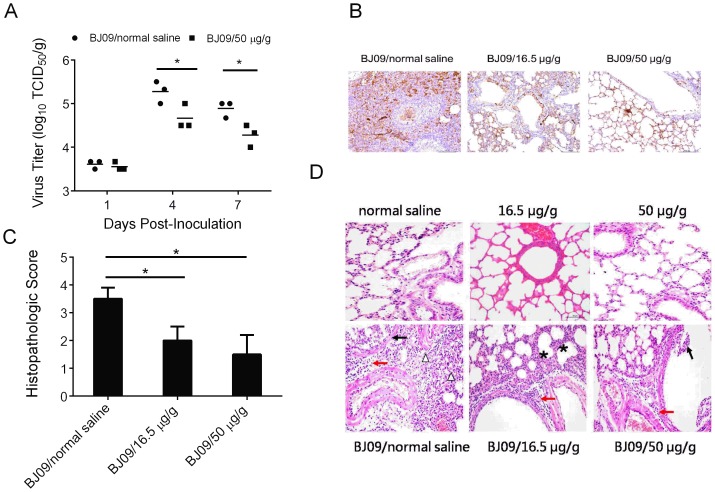
*Cryptoporus volvatus* extract inhibits H1N1 influenza virus replication *in vivo*, and reduces severity of pathological changes. (A) Virus replication in lung of mice. Titers of virus recovered from the supernatant of homogenized lung at day 1, 4, and 7 p.i. are shown. (B) Immunohistochemistry analysis of the lung of influenza virus-infected mice treated with normal saline or the extract. Scale bars, 100 µm. (C) Lung histological grading of virus-infected mice treated with normal saline or the extract (5 sections from each lung, and 3 mice per group). (D) Representative of histopathological changes in H&E stained lung tissues from mice sacrificed at day 4 p.i.. normal saline (negative control group); 50 µg/g (extract control group), no histopathology lesion; BJ09/normal saline (virus infection control group): severe desquamation and droplet of bronchial mucosa (↑), massive immune cell and red blood cell infiltrates around bronchi and blood vessels (red arrow), and inflammatory cells within alveolar spaces (△); BJ09/16.5 µg/g (virus infection and treated with low-dosage extract group): a small number of immune cell infiltrates around bronchi and blood vessels (red arrow), and alveolar wall thickened (*); BJ09/50 µg/g (virus infection and treated with high-dosage extract group): a small number of immune cell infiltrates around bronchi and blood vessels (red arrow), and mild desquamation of bronchial mucosa (↑). (Scale bar: 50 µm). Data are presented as mean ±SD. Statistical significance was analyzed by *Student*'*s t test*; *P<0.05; **P<0.01.

Previous study demonstrated that H1N1/09 virus was restricted to the respiratory systems of mice and replicated most efficiently in lungs [28]. To examine the ability of the *Cryptoporus volvatus* extract to inhibit H1N1/09 virus replication in mice, we collected the lungs of three mice in two of the three treatment groups to quantify H1N1/09 virus at day 1, 4, and 7 post infection (Fig. 4A). H1N1/09 virus replicated efficiently in the lungs of mice from saline treatment group with a titer of 10^5.27^ TCID_50_/g. In comparison, the viral burdens in high-dosage drug treatment group were about 4- fold lower than that in no treatment control group at 4 days post infection (P<0.01). Similar results were also observed at 7 days post infection. These findings indicated that the *Cryptoporus volvatus* extract treatment was capable of suppressing H1N1/09 virus replication in mice. We further investigated influenza virus distribution in lungs by IHC (Fig. 4B). In virus-infected mice treated with normal saline, the bronchiolar wall was ulcerated and there was extensive and intense viral antigen staining in bronchiolar epithelium, which was sloughing into the lumen. There was also viral antigen staining in the alveolar and staining of cells in the peribronchiolar inflammation areas. The level of influenza virus-positive cells in the extract-treated group, especially in the high concentration group, was lower than that in the lungs of normal saline-treated mice, and viral antigen staining appeared to be limited to the bronchial epithelium and minimal in the interstitial epithelium (alveolar septa). These results suggest that the extract could decrease influenza virus distribution in the lungs.

We also examined the ability of the *Cryptoporus volvatus* extract to prevent virus infection- induced pulmonary lesions at day 4 following H1N1/09 infection. As shown in Fig 4C and 4D, H&E staining showed that H1N1/09 virus caused severe interstitial pneumonia and bronchopneumonia characterized by serious dropout of bronchial mucosa and extensive infiltration of inflammatory cells and red blood cells in bronchia and alveolus (Fig. 4C and D, BJ09/normal saline). In comparison, less severe pneumonia was observed in animals treated with low-dosage *Cryptoporus volvatus* extract (Fig. 4C and D, BJ09/16.5 µg extract/g body), while pathology observed in animals of high-dosage treatment group was milder (Fig. 4C and D, BJ09/50 µg extract/g body).

### *Cryptoporus volvatus* extract protects mice from virus challenge

**Figure 5:**
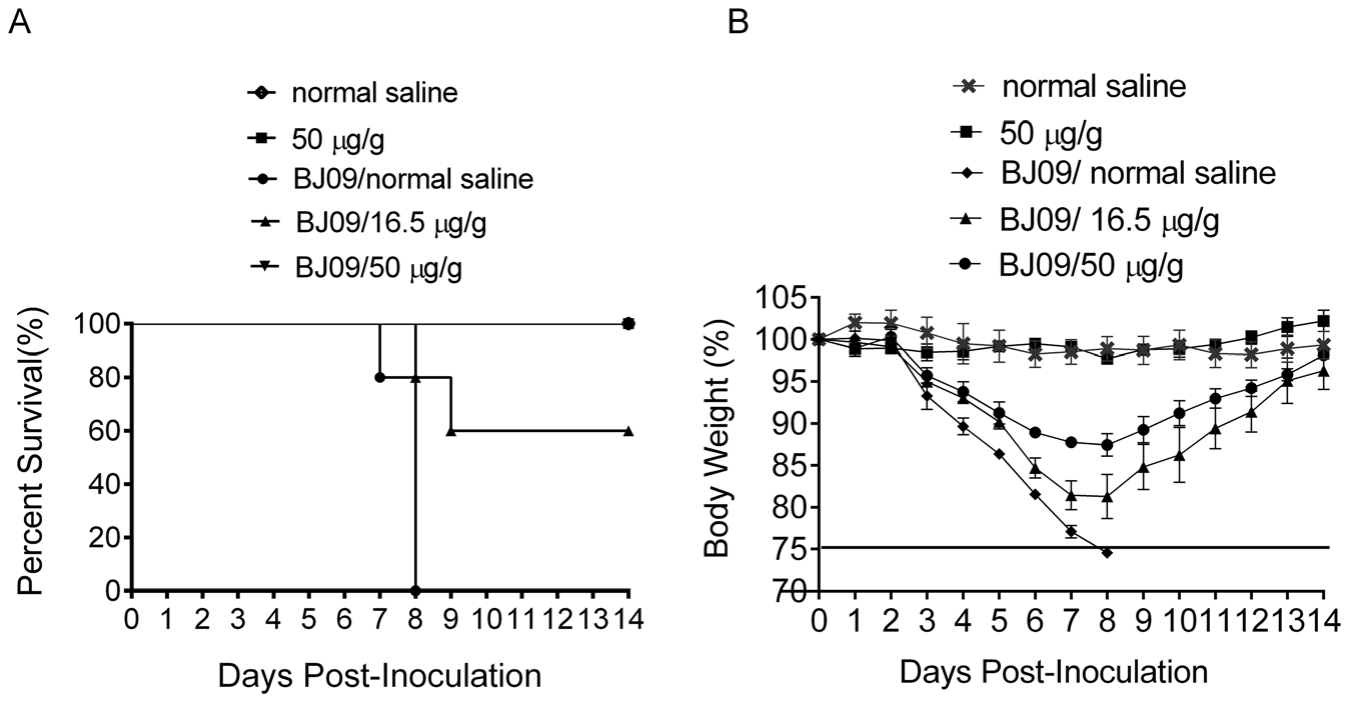
Characterization of *Cryptoporus volvatus* extract efficacy in a mouse model of H1N1/09 influenza virus infection. (A) Survival rate and (B) Weight changes of mice. The dashed line indicates 75% of initial body weight, and data are presented as mean ±SD. Mice infected with or without H1N1/09 influenza virus were treated with the extract or with saline. Each group contained five BALB/c mice. Body weight and survival status were checked daily. Mice were euthanized upon the loss of 25% of their initial body weight. normal saline (no virus as negative control group); 50 µg/g (no virus as extract control group); BJ09/normal saline (virus infection (10^3.5^ pfu) control group); BJ09/16.5 µg/g (virus infection (10^3.5^ pfu) and treated with low-dosage extract group); BJ09/50 µg/g (virus infection (10^3.5^ pfu) and treated with high-dosage extract group). Data are presented as mean ±SD.

H1N1/09 influenza virus could cause mice lethal disease. To determine whether the *Cryptoporus volvatus* extract can protect mice from H1N1/09 influenza virus lethal challenge, we treated H1N1/09 infected mice with low-dose or high dose of the *Cryptoporus volvatus* extract for 8 days. By day 4 p.i., most of the mice infected with H1N1/09 virus in saline treatment group showed severe clinical signs of respiratory disease, including labored respirations and respiratory distress. All five mice died from 7 to 8 day p.i (Fig. 5A). Mice exhibited unable response to exterior stimuli, polypnea, and labored respirations before death. The mortality of infected mice was reduced following low-dosage *Cryptoporus volvatus* extract treatment (2/5, 40%). However, mice still showed obvious clinical signs, including decreased activity, huddling, hunched posture, and ruffled fur. Strikingly, high-dosage of the *Cryptoporus volvatus* extract treatment prevented all 5 mice from death. Mice in this group did not show obvious clinical signs except for slight weight loss (Fig. 5B). Mice in normal saline control and extract control groups did not display any clinical signs during the course of the experiment. Taken together, these data suggested that the *Cryptoporus volvatus* extract could inhibit H1N1/09 influenza virus replication, and protect mice from H1N1 influenza virus infection.

## Discussion

Influenza viruses are still the cause of significant morbidity and mortality, posing a serious health threat during seasonal outbreaks as well as periodic pandemics, although influenza vaccines and two classes of anti-influenza virus drugs are available. Thus, there is an urgent need to develop new regimens.

Chinese herbal medicines are a unique source of medical complexity and diversity, and they have been exploited extensively in pursuit of new antiviral agents [12]. *Cryptoporus volvatus* has a long medical use history for treating asthma and bronchitis in China [22]. We previously reported that the aqueous extract from the fruiting body of *Cryptoporus volvatus* has the potential to inhibit porcine reproductive and respiratory syndrome virus (PRRSV) infection *in vivo* and *in vitro*. In this study, we found that the aqueous extract from the fruiting body of *Cryptoporus volvatus* also had broad and robust activity against influenza virus infection. Our data demonstrated that the extract could inhibit different influenza virus strain infection. And most importantly, we showed that the extract completely protected mice from lethal challenge with H1N1/09 influenza virus in a mouse model when we treated the mice with a high dose of the extract.

We first investigated the potential of the *Cryptoporus volvatus* extract to inhibit influenza virus infection i*n vitro*, and its toxicity on cells. We showed that the CC_50_ of the *Cryptoporus volvatus* extract for MDCK cells was 148 mg/ml, which greatly exceeded its EC_50_ (0.45 mg/ml for H1N1/09 strain, 1.21 mg/ml for H3N2 strain and 0.37 mg/ml for A/WSN strain). The therapeutic index (CC_50_/EC_50_) in MDCK cells was 328 for H1N1/09 influenza virus, 68 for H3N2 virus, and 400 for A/WSN virus strain.

Then we investigated on which step(s) of influenza virus life cycle the extract exerted its effect to inhibit virus infection. The inhibitory effect of the *Cryptoporus volvatus* extract is not due to its direct inactivation of H1N1 virion particles, as its incubation with A/WSN (H1N1) virus at 37°C for 1 h or 3 h had no effect on viral infectivity (Fig. 2B). In the time-of-addition experiments, the extract lost partial anti-influenza virus activity when added at 1 h p.i. and still had the comparable inhibition activity when added at 8 h p.i, indicating that its target is situated at both the earlier and late stages of virus replication. Earlier event during influenza virus infection is virus entry into cells, which is consisted of virus binding to cells and internalization, virus uncoating, release of the vRNP in the cytoplasm, and importing of the vRNP into the nucleus [34]. Our virus binding experiments at 4°C provided evidence that the extract did not have inhibitory effect on receptor-mediated virus binding. However, using time of addition experiment, we demonstrated that cellular uptake of the virus was significantly blocked by the extract. Entry process is attractive as targets to block infection efficiently as it is the first essential step for virus replication. The acute nature of influenza virus infection and the accompanying inflammatory disease also make an intervention strategy by blocking the early viral entry process particularly favorable [35]. This is consistent with our previous study that the *Cryptoporus volvatus* extract blocks PRRSV entry into cells. Both viruses use similar entry routes and so it is conceivable that the extract maybe target cellular factor(s) which is essential for virus entry. However, as the extract is a mixture of many chemical compounds, we could not exclude the possibility of targeting viral proteins that mediate virus entry into cells, e.g. the receptor binding protein haemagglutinin (HA). Further studies are needed to illustrate the mechanism by which the extract inhibits virus entry.

More importantly, in the animal study, we found that the extract could completely protected mice from lethal challenge with H1N1/2009 influenza virus. To make sure that the dose we used in mouse study did not cause much direct damage to the mice, the highest dose we used was 50 µg/g body weight. Indeed, the extract alone at this dose did not cause obvious weight loss and lung lesions compared to the saline/no virus control. To our surprise, the extract at high dose (50 µg/g body weight) not only reduced virus loads in lungs of mice, but also protected mice from lethal challenge with H1N1 influenza virus. These results suggest that the *Cryptoporus volvatus* extract has the potential to be used to treat H1N1 influenza virus infected mice. However, we could not exclude the fact that the H1N1 influenza virus infected mice treated with high dose of the extract still lost some weight compared to saline/no virus controls, even though these mice survived. Even though we showed that *Cryptoporus volvatus* could significantly inhibit H1N1 influenza virus replication in lungs of mice, the reduction of virus titre was minimal. We propose that there might be other mechanisms existed for *Cryptoporus volvatus* to protect mice from H1N1 influenza virus lethal challenge, e.g. suppressing inflammation. Indeed, *Cryptoporus volvatus* is used to treat bronchitis [22]. Thus, more works remain for us to do. Aqueous extract from the fruiting body of *Cryptoporus volvatus* is a crude extract, which includes many components. The antiviral effects of the extract might result from a mixture of active compounds rather than from a single chemical entity. The efficacy of Traditional Chinese Medicine (TCM) is a characteristic of a complex mixture of chemical compounds present in the various herbs. The concept of combinatorial medicines has been exemplified by the drug cocktail used in the treatment of acquired immunodeficiency syndrome [36]. However, in order to develop new generation of antiviral agents, it is necessary to isolate and purify the active compounds in the aqueous extract from the fruiting body of *Cryptoporus volvatus*. Obviously, more work remains for us to do to identify molecules in the *Cryptoporus volvatus* extract.

## Conclusions

In conclusion, our findings reveal that the aqueous extract of *Cryptoporus volvatus* exhibits antiviral activity against influenza A virus *in vitro* and *in vivo*, and has the potential to be developed into a new antiviral agent. Further studies are in progress to identify the molecules that are responsible for the inhibitions of virus replication.
